# Effect of home-based transcranial direct current stimulation (tDCS) on cognitive function in patients with mild cognitive impairment: a study protocol for a randomized, double-blind, cross-over study

**DOI:** 10.1186/s13063-019-3360-1

**Published:** 2019-05-21

**Authors:** Jaesub Park, Yoonkyung Oh, Kyungmi Chung, Kwang Joon Kim, Chang Oh Kim, Jin Young Park

**Affiliations:** 1 Department of Psychiatry, National Health Insurance Service Ilsan Hospital, Goyang, South Korea; 2 Department of Psychiatry, Gangnam Severance Hospital, Yonsei University Health System, 211 Eonju-ro, Gangnam-gu, Seoul, 06273 South Korea; 3 Department of Psychiatry and Institute of Behavioral Science in Medicine, Yonsei University College of Medicine, Seoul, South Korea; 4 Division of Geriatrics, Department of Internal Medicine, Severance Hospital, Yonsei University College of Medicine, Seoul, South Korea

**Keywords:** Transcranial direct current stimulation, Mild cognitive impairment, Home-based, Cross-over study, Brain stimulation

## Abstract

**Background:**

The possible effect of transcranial direct current stimulation (tDCS) in improving cognitive function is clear from studies involving pre-dementia stage mild cognitive impairment (MCI). However, the application of tDCS in actual clinical practice entails repeated hospital visits almost every day for treatment. The objective of this study is to confirm the possibility of self-application of tDCS at home by elderly patients with declined cognitive function and the significant clinical effect of tDCS administered at home.

**Methods/design:**

This study will be conducted in 20 elderly people aged 60 to 80 years with complaints of subjective memory impairment while maintaining general functions with limited activities of daily living. This study involves a cross-over design that will include 2-week active or sham stimulation of both dorsolateral prefrontal cortexes (left, anode; right, cathode) randomly with a 2-week wash-out phase. Changes in cognitive function will be evaluated using visual recognition tasks and neuropsychological tests. In this study, tDCS will be carried out by each patient at his/her home and its safety and suitability will be evaluated.

**Discussion:**

In this study, patients will apply a portable tDCS, developed for home use, for more than 2 weeks. Such studies can contribute to the use of tDCS as a realistic therapy. In addition, the utility of home-based tDCS will be confirmed by application of tDCS at home by the elderly with declined cognitive function. Furthermore, confirmation of tDCS as a significant therapeutic method can facilitate treatment of Alzheimer’s dementia at an early stage, including MCI.

**Trial registration:**

Clinical Research Information Service (CRIS), KCT0002721. Registered on 9 March 2018.

**Electronic supplementary material:**

The online version of this article (10.1186/s13063-019-3360-1) contains supplementary material, which is available to authorized users.

## Background

Mild cognitive impairment (MCI) refers to declined cognitive function below normal range while maintaining successful independent daily living [1]. Declined cognitive function in MCI falls between cognitive deterioration caused by aging and dementia due to Alzheimer’s disease or other [2]. MCI might be a precursor to dementia [3], which is an important topic for clinical trials investigating dementia treatment and prevention. Transcranial direct current stimulation (tDCS) is a therapeutic modality indicated for declined cognitive function, including MCI.

tDCS is used to control neuronal excitability by transmitting a small amount of current via an electrode patch on the scalp. Anodal stimulation increases cortical activity by drawing the resting potential close to the threshold potential while cathodal stimulation inhibits excitability by separating the resting potential from the threshold potential [4]. As control over brain activity affects brain functions such as behavior, emotion, and information processing, clinical application of tDCS for depression, multiple sclerosis, and other central nervous system disorders is under investigation by several clinical groups [5–7].

The effect of tDCS on cognitive function has been established in various clinical trials and experimental studies. In a study conducted in healthy adults, visuospatial working memory performance status improved after anodal tDCS [8]. Another study has shown that tDCS can result in temporary but meaningful improvement in aging-related cognitive decline [9]. In tDCS and repeated transcranial magnetic stimulation (rTMS) studies conducted in individuals with declined cognitive function, improvement of cognitive function has been reported [9–13]. In a study by Meinzer et al. [11] in particular, anodal tDCS increased task performance and reversed pathological brain activity and connectivity patterns. Boggio et al. [13] confirmed that the performance in visual memory recognition task is improved after tDCS treatment of dorsolateral prefrontal cortex in a study of patients with Alzheimer’s dementia. In these studies, tDCS has been applied temporarily or without sufficient repetition. Therefore, it is necessary to evaluate the effect of repeated tDCS sessions on cognitive function in patients with MCI.

tDCS is user-friendly, relatively cheap, and tolerable compared to similar non-invasive brain stimulating devices such as rTMS [14]. In addition, no serious side effects have been reported with tDCS, thus encouraging home use [15]. In fact, home-based clinical application of tDCS has been investigated in a number of studies in depression, chronic pain, multiple sclerosis, tinnitus, and mild vascular dementia [16–20]. However, to the best of the author’s knowledge, home-based clinical application of tDCS has not been investigated in MCI. Furthermore, the same equipment used in hospitals is also used in previous home-based tDCS studies. Therefore, the development of smaller, low-weight, and automated devices and additional studies investigating the suitability and safety of home-based application are needed.

This study will investigate the effect of tDCS on cognitive function and the safety of self-application by instructing patients with MCI to self-administer tDCS repeatedly using the device developed for home-based application. This study is designed to determine whether repeated tDCS stimulation at home can improve cognitive function by visual recognition task and neuropsychological tests in patients with MCI. It will also determine whether patients with MCI can safely administer tDCS by themselves at home.

## Methods/design

This study will involve patients diagnosed with MCI who visited Gangnam Severance Hospital and Severance Hospital, tertiary referral centers and the teaching hospital of Yonsei University. All participants will be informed of experimental goals of this study. Written informed consent will be obtained. This study protocol was approved by the Korean Ministry of Food and Drug Safety and complied with ethical standards based on Declaration of Helsinki.

### Inclusion criteria

Selection criteria are: 1) adults aged between 60 and 80 years; and 2) patients with cognitive decline from previous performance levels but which do not interfere with capacity for independence in everyday activities. Cognitive decline but not dementia will be determined using the Korean version of the Global Deterioration Scale (GDS; 2–3 points) [21] or Clinical Dementia Rating (CDR; if 0.5) and MMSE-DS (Korean version of MMSE for Dementia Screening; higher than the cut-off according to age and academic background) [22]. Independence in activities of daily living will be determined according to Seoul-Instrumental Activities of Daily living score [23] (< 7 points). Complaints of cognitive decline induced by depression will be evaluated using K-GDS (Korean version of Geriatric Depression Scale) (≤ 8 points) [24]. Final clinical diagnosis will be conducted by skilled psychiatrists using DSM-V (Diagnostic Statistical Manual of Mental Disorder, fifth edition) criteria for mild neurocognitive disorder.

### Exclusion criteria

Exclusion criteria are as follows: 1) history of dementia or intellectual disability; 2) use of cognitive enhancers (donepezil, rivastigmine, galantamine, and memantine); 3) reversible medical conditions associated with potential cognitive impairment (e.g., history of vitamin B12 deficiency or hypothyroidism); 4) diagnosis of current alcohol dependence or DSM-V [25] disorders associated with the use of alcohol; 5) epilepsy; 6) clinically severe disorders involving cardiovascular, digestive, respiratory, endocrine, and central nervous systems; 7) history of cerebrovascular surgery; 8) problems associated with direct current stimulating electrode caused by scalp malformation, inflammatory reaction, or other dermatological problem; and 9) other contraindications for tDCS medical device (e.g., metal plate inserted into cephalus, etc.). In addition, patients who participated in other clinical studies within the past 30 days, who expressed difficulty in reading or having a conversation due to vision and hearing issues after wearing assistance, and those who are ineligible for this study based on other medical judgment will be excluded from this study. Use of other psychiatric medication except cognitive enhancers and general medications will be permitted.

### Outcome measures

#### Primary outcomes

This study will determine cognitive improvement by using visual recognition tasks similar to those described previously [12, 13] in which tDCS is administered to patients with Alzheimer’s dementia. In the encoding phase, screens with two, four, six, and eight stimulations are displayed for 10 s, respectively. Participants are required to memorize pictures displayed on each screen. After a 1-s interval, the test phase is started and the specific stimulus is displayed. Participants are required to respond as rapidly as possible whether or not the stimulus involves the picture seen in the encoding phase. Similar to previous studies, our task comprises an encoding phase and a test phase. In contrast to previous studies, black-and-white icon stimuli will be used to provide a more intuitive and simple format. Objects, human body parts, and animals that are generally seen in daily life will be used. The test phase will comprise three, six, eight, and ten test trials of two, four, six, and eight pictures, respectively, in the encoding phase.

#### Secondary outcomes

In addition to visual recognition tasks, standardized neuropsychological tests and questionnaires will be used to determine cognitive improvement and changes in daily life, which will be assessed by 1) letter-number sequencing; 2) spatial span; 3) clinical global impression (CGI) [26]; 4) neuropsychiatric inventory [27]; and 5) Korean version of the Consortium to Establish a Registry for Alzheimer’s Disease Neuropsychological Assessment Battery (CERAD-K) [28].

#### Covariate measures

Additionally, a questionnaire will be used to determine factors that influence the therapeutic utility of tDCS. The questionnaire includes the following: 1) Edinburgh Handedness Inventory [29]; 2) State Anxiety Inventory (STAI-X-1) [30, 31]; 3) Montgomery-Asberg Depression Rating Scale (MADRS) [32, 33]; 4) Korean version of the Hamilton Depression Scale (K-HDRS) [34]; and 5) Hamilton Anxiety Scale (HAM-A) [35].

### Intervention

#### tDCS stimulation protocol

The electrode will be located by placing the anode and cathode on the left dlPFC (F3) and the right dlPFC (F4), respectively, similar to the procedure described by Boggio et al. [13]. To facilitate placement of the electrode at home, a skilled investigator will provide a picture of a desirable location taken with a smart phone. Similar to previous studies [12, 13, 36], active stimulation will increase the current for 30 s. The current is then kept at 2 mA for 29 min. The current is then decreased for 30 s. Sham stimulation will be maintained for 29 min without current flow by increasing current for 30 s followed by a decrease for 30 s. Both conditions will be self-administered by the subject for 30 min each day for 14 consecutive days.

#### Home-based tDCS device

**Fig. 1:**
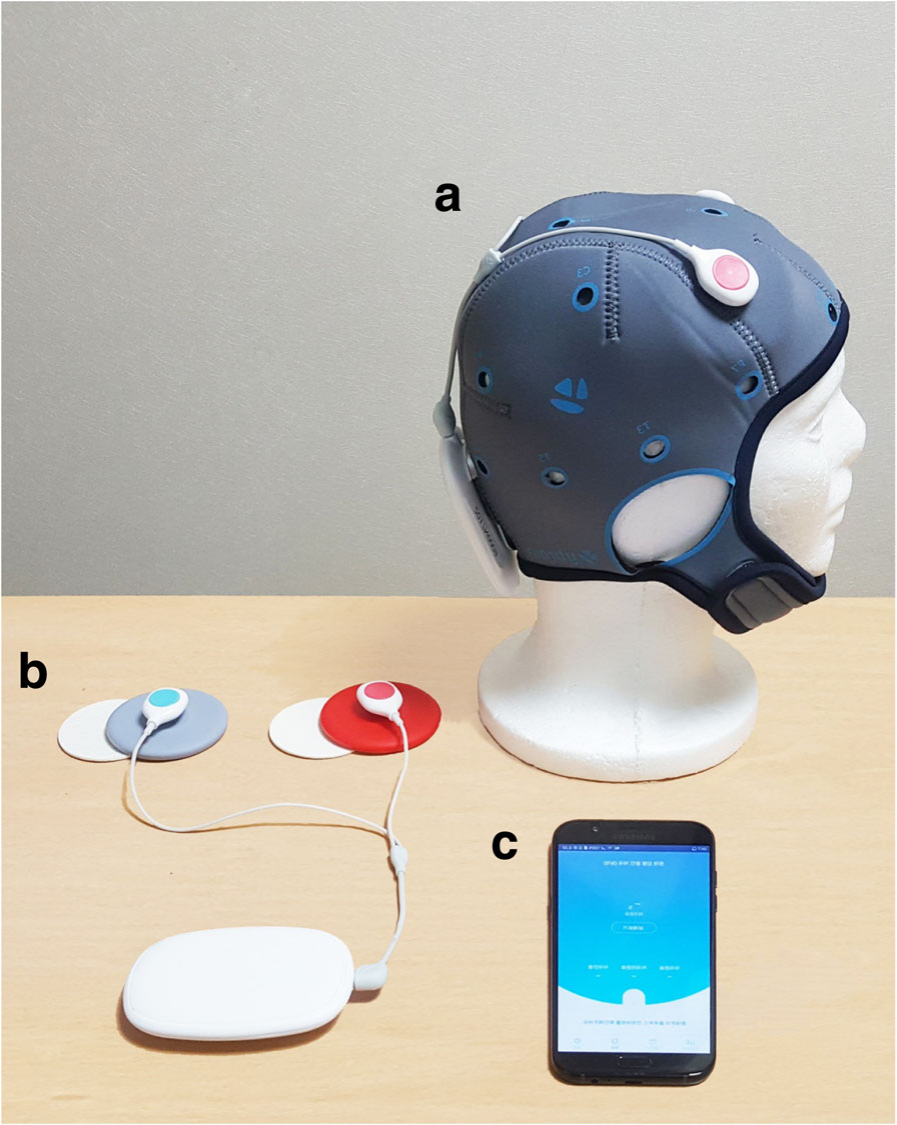
Image of the device provided to study participants. **a** Module and electrode while wearing cap. **b** tDCS module and electrode. **c** Smart phone with application

This study will be conducted using a tDCS device (Ybrain, MINDD STIM, http://www.ybrain.com/) designed for application at home. The device consists of 1) a docking station for stimulation setup and information output, 2) a miniaturized module for saving the performance record that is compatible with a smart phone, and 3) a smart phone. The module works with any smart phone, although we will use a research smart phone to control the experimental environment. The module transfers the stimulation performance records to a server linked to the docking station via a compatible smart phone that can be remotely monitored by the investigator. The electrode of the device, which is circular in form and measures 67 mm in diameter (or 22 mm thick) will be used after inserting a disposable sponge into the patch supporter followed by treatment with saline solution. The electrode will be inserted into the pre-selected hole of the cap for fixing on the head and linking to the module (Fig. 1).

#### Safety strategy for home-based tDCS application

Several technologies will be used to safely conduct tDCS independently at home. The stimulation protocol is not modifiable in the module by the subject as it is accessible only with a password via the station. The module contains a skin-contact recognition function. Therefore, the device automatically starts upon recognition of the patch adhering to the skin. The output automatically stops upon recognition of the patch being detached from the skin, even during stimulation. The device stops immediately when the current output exceeds the limit allowed by the module. A smart phone application detects module operation. A self-inspection questionnaire for safety assessment will be administered upon completion of the operation. In addition, 24-h contact information is provided via the smart phone. In case of any suspected side effects, the subject is expected to visit the hospital immediately.

#### Training and support for participants

Even though the device has a safety guarantee, it might be meaningless if the user is not familiar with its usage. Therefore, checklist-based training will be conducted by using the device for practice without stimulation. Subjects who have completed all items of the checklist will be allowed to use the device for the study after three training sessions and the final test. Remote support will be provided via a remote-control program integrated in the smart phone and video calling will be used to facilitate the study.

### Design and procedure

#### Trial design

**Fig. 2:**
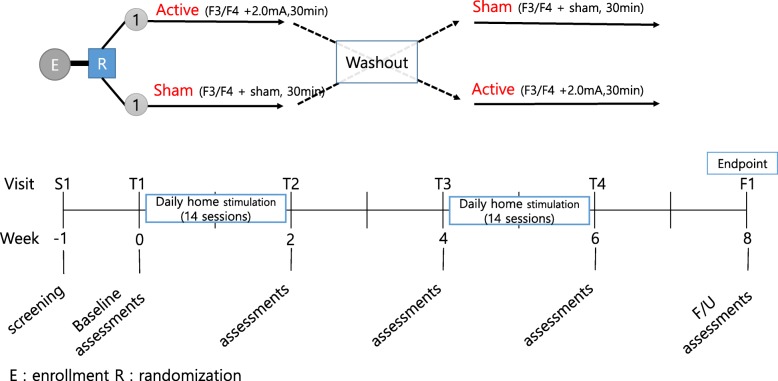
Study design and method

This study was designed as a 9-week, multicenter, randomized, double-blinded, cross-over clinical study. All subjects will undergo a 2-week treatment phase twice (treatment I and treatment II) with a 2-week washout period. A follow-up assessment will be conducted 2 weeks after completion of the second treatment phase (treatment II; Fig. 2). In treatment I, either active or sham tDCS will be administered. In treatment II, tDCS under different conditions will be administered.

**Fig. 3:**
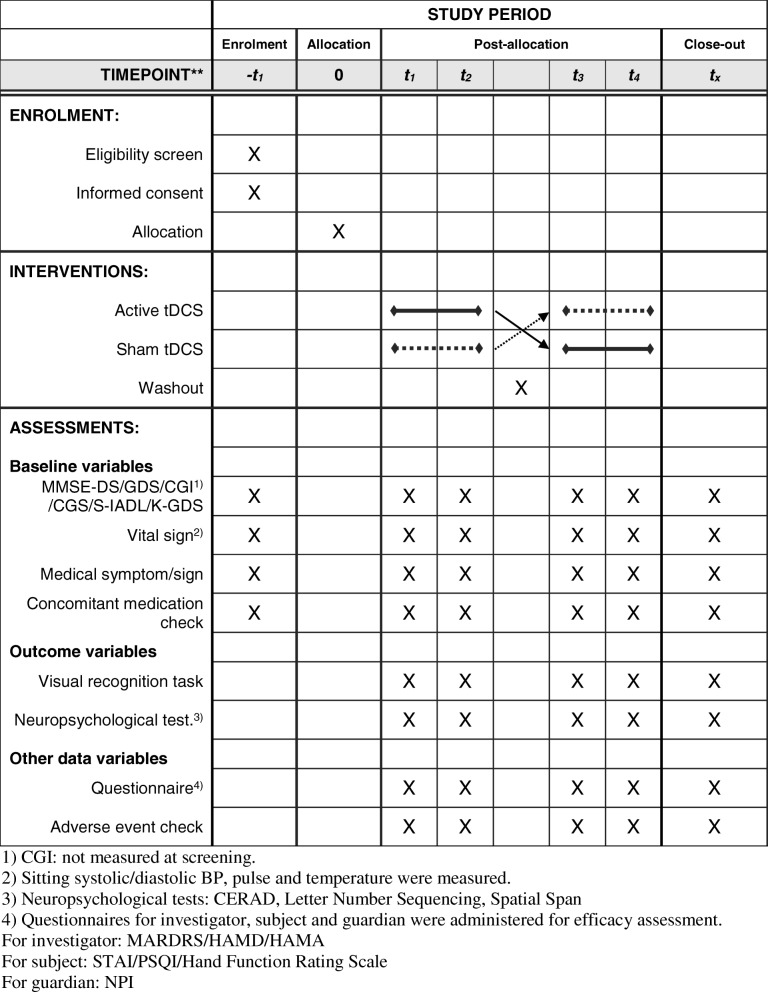
Schedule of enrolment, interventions, and assessments

#### Randomization and blinding

A random group assignment list was created by statisticians who are not involved in data collection using PASS 12 (NCSS, LLC, Kaysville, Utah, USA, https://www.ncss.com/). Participants are allocated 1:1 to the active first group and sham first group. The device manager will set up the device according to the assignment list in order of participant’s registration number. The same single device can be set to sham or active status using the administration menu. Because only the device manager, who will not contact the participants, knows the assignment list and the password required to access the administration menu, other investigators and participants will remain blind to it. This masks investigators and enables the use of fewer devices.

#### Study schedule

Assessments at each visit are listed in Table 1 & Fig. 3. Details of each phase are as follows:

1. Screening phase: Only participants with written consent will be screened. Subjects who meet the inclusion criteria will be randomized.
2. Treatment phase I (visit 1 to visit 2): During visit 1 (T1), a visual recognition task and a neuropsychological test will be conducted with baseline data for efficacy assessment. After the outcome measures are taken, including covariance measurements, training for tDCS use will be conducted for subjects or their caregivers by using a tDCS device for practice and a check list. The first clinical device application and assessment of side effects will be conducted for subjects who have passed the test for tDCS use. Starting from the day after the first visit, the subject (or his/her caregiver) will use the device once daily for 2 weeks (a total of 14 days). It is recommended to use the device at specific times (e.g., immediately after face washing in the morning). Information about time and period of application by the subject are automatically saved in the device. Researchers will monitor the information remotely at certain times of the day and will call if tDCS is not applied.
3. Wash-out phase (visit 2 to visit 3): The subject will be required to visit the hospital 2 weeks after visit T1 for efficacy assessment. Subsequently, appropriate use of the device by subjects will be assessed by the investigator using a tDCS device for practice. A log of the returned device will be printed by the device manager for records so that blinding is maintained. Later, a 2-week wash-out phase will be conducted.
4. Treatment phase II (visit 3 to visit 4): The subject will be required to visit the hospital at 4 weeks after visit T1. Similar to treatment I, training sessions will be conducted and proficiency with the device will be assessed. Also similar to treatment 1, the device will be used at home after returning from the hospital. After returning home, subjects will apply the device once daily for 2 weeks (14 days).
5. Follow-up phase: Subjects will be asked to visit the study institution at 6 weeks after visit T1. Appropriateness of device application and efficacy assessment will be evaluated by the investigator. Finally, a follow-up assessment will be conducted during a study visit at 2 weeks after the last treatment visit (T4).

**Table 1:** List of tests conducted at each visit

Phase	Screening	Treatment I	Washout	Treatment II	Follow-up	
Day	0	1	14	28	42	56
Visit	S1	T1	T2	T3	T4	F1
Visual recognition task		O	O	O	O	O
Neuropsychological test^a^		O	O	O	O	O
Basal symptom and sign	O	O	O	O	O	O
Vital sign^b^	O	O	O	O	O	O
MMSE-DS/GDS/CGI^c^/CGS/S-IADL/K-GDS	O	O	O	O	O	O
Questionnaire^d^		O	O	O	O	O
Concomitant medication check	O	O	O	O	O	O
Adverse event check		O	O	O	O	O

^a^Neuropsychological tests: CERAD, letter number sequencing, spatial span

^b^Sitting systolic/diastolic blood pressure, pulse, and temperature were measured

^c^CGI: not measured at screening

^d^Questionnaires for investigator, subject, and guardian were administered for efficacy assessment

For investigator: MARDRS/HAMD/HAMA

For subject: STAI/PSQI/Hand Function Rating Scale

For guardian: NPI

### Statistical analysis

#### Sample size

The sample size was calculated based on results reported by Boggio et al. [12]. Boggio et al. [12] administered active or sham tDCS once daily for 5 days to patients with Alzheimer’s dementia and compared their performance for visual recognition tasks before and at 1 month after treatment. Based on differences in mean correct response rate (ε = 11.61, σ_d_ = 11.62; 1:1 allocation ratio per group) in the previous study, a sample size was estimated to obtain at least 90% power (1 − beta = 0.9) to detect a treatment difference of 11.61 (SD 11.62 of change) in visual recognition task scores at a significance level of 0.05 (α = 0.05). A total of 14 persons (seven per sequence) were selected by PASS 12 (NCSS, LLC, Kaysville, Utah, USA, https://www.ncss.com/) as the smallest sample size. This study targeted a total of 20 subjects (10 per group) considering a drop rate of 30%.

#### Statistical methods

For primary efficacy assessment, differences in correct response rate in the visual recognition task before and after treatment will be calculated. Differences between active and sham setups will be analyzed by a general linear model [37] to assess major (treatment), period (time), and carry-over effects. For secondary efficacy assessment, analysis of variance (ANOVA) of changes in each sub-item of CERAD-K will be conducted. Only the results of the participants who completed the intervention will be included in these efficacy analyses, but the side effects will be reported for all participants.

## Discussion

Despite promising results reported by studies investigating cognitive improvement using non-invasive brain stimulation methods, their actual use in clinical practice is still limited. Reasons restricting their clinical use may include: 1) the long-term effect cannot be confirmed as most previous studies only monitored changes after single and temporal treatment; 2) although the effect of > 2-week repetitive application was observed in several studies, patients were asked to visit hospital every day in order to reproduce such effects in actual practice. In this study, patients will be asked to use miniaturized, automated tDCS that is available for self-administration at home under remote surveillance. If the elderly with cognitive decline can use tDCS at home, patients with diverse diseases without cognitive impairment can use tDCS safely as well. Our findings may suggest that home-based tDCS is a practical treatment for patients, thus addressing limitations of non-invasive brain stimulation methods described previously.

Previous results have indicated a high incidence of dementia in elderly patients with MCI compared to the general elderly population. According to Petersen et al. [1, 3], approximately 10–15% of the aging population progresses to Alzheimer’s disease while 80% of elderly with MCI present symptoms of dementia after 6 years. However, drug therapies that delay progress from MCI to dementia or improve cognitive function are unknown [38]. Considering that the elderly are generally vulnerable to side effects of drugs and drug–drug interactions due to polypharmacy, non-drug therapies are in demand. The potential of tDCS to significantly improve cognitive function in MCI provides a significant treatment alternative for not only patients with MCI, but also those with early-stage Alzheimer’s dementia. In addition, improving cognitive function is expected to help reduce social care burdens and improve patients’ subjective quality of life by maintaining their independence.

Cognitive function consists of various interacting domains, with specific domains influenced greatly by disease or therapy. This study seeks to use visual recognition tasks as well as standardized comprehensive neuropsychological assessments and symptom questionnaires for efficacy assessment. Therefore, it is expected to unravel complex patterns of change in cognition and symptoms related to the use of tDCS. In addition, it may facilitate the assessment of neuropsychological characteristics of patients with significant response to tDCS.

Lastly, applications and technologies for symptom and side effect assessment as well as remote support that are integrated into the smart phone in this study are applicable not only to tDCS studies, but also to other clinical trials. Such home-based clinical trial systems utilizing established technologies are inexpensive to install. They are highly expandable due to compatibility with a wide range of devices and applications. These methodologies are relevant to elderly patients facing difficulty with hospital visits. They are also useful for clinical studies investigating the effect of physical activity using ambulatory monitoring devices.

This study has some limitations. First, the study is a small-scale cross-over study. Although cross-over design enables exploratory studies in a relatively small number of patients, a large-scale study is needed to validate the clinical effect. Second, a 2-week wash-out period in this study might be insufficient given that studies demonstrating a consistent effect with tDCS are still unavailable. The effect of tDCS has been reported to continue in patients with Alzheimer’s disease [12]. To overcome this limitation, we will analyze the carry-over effect using a general linear model. Third, psychological factors in the elderly associated with the use of a smart phone may influence this study. The elderly who experience cognitive decline may feel burdened by having to learn how to use the new devices. This can influence their compliance with the study protocol. The interface and devices should be optimized for use in the elderly population and verified in further studies.

In summary, this study will facilitate the application of tDCS in MCI as a home-based, self-administered intervention and a realistic therapy. Furthermore, confirmation of tDCS as a significant therapeutic method will facilitate treatment for Alzheimer’s dementia at an early stage, including MCI (Additional file 1).

## Trial status

Recruitment of participants started in March 2018 and will be completed in July 2019. The manuscript reports protocol version 1.1 (November 24, 2017).

## Additional file

Additional file 1: SPIRIT 2013 checklist: Recommended items to address in a clinical trial protocol and related documents. (DOC 122 kb)

## Abbreviations

ANOVA: Analysis of variance
CDR: Clinical Dementia Rating
CERAD-K: Consortium to Establish a Registry for Alzheimer’s Disease Neuropsychological Assessment Battery
CGI: Clinical Global Impression
HAM-A: Hamilton Anxiety Scale
K-GDS: Korean Version of Geriatric Depression Scale
K-HDRS: Korean version of the Hamilton Depression Scale
MADRS: Montgomery-Asberg Depression Rating Scale
MCI: Mild cognitive impairment
MMSE-DS: Korean version of MMSE for Dementia Screening
rTMS: Repeated transcranial magnetic stimulation
STAI-X-1: State Anxiety Inventory
tDCS: Transcranial direct current stimulation
